# De Novo Lipogenesis in Clear Cell Renal Cell Carcinoma: Mechanistic Insights and Therapeutic Implications

**DOI:** 10.3390/ijms27114924

**Published:** 2026-05-29

**Authors:** Kha Cong Le Bui, Yen Thi Do, Jin Young Kim, Ji Hae Seo

**Affiliations:** 1 Department of Urology B, Hue Central Hospital, Hue 49135, Vietnam; drkhabui.urologist@gmail.com; 2 Division of Haematology and Oncology, Department of Internal Medicine, School of Medicine, Keimyung University, Daegu 42601, Republic of Korea; 3 Department of Biochemistry, School of Medicine, Keimyung University, Daegu 42601, Republic of Korea; yen94d@gmail.com

**Keywords:** de novo lipogenesis, clear cell renal cell carcinoma, VHL–HIF signaling, metabolic reprogramming, lipid reprogramming, lipogenesis inhibitors

## Abstract

Clear cell renal cell carcinoma (ccRCC) is increasingly recognized as a lipid-addicted malignancy in which de novo lipogenesis (DNL) supports tumor growth, survival, and treatment resistance. ccRCC-specific genetic alterations, particularly loss of VHL and activation of hypoxia-inducible factor (HIF) signaling, promote SREBP-mediated upregulation of key lipogenic enzymes, including ATP-citrate lyase (ACLY), acetyl-CoA carboxylase (ACC), fatty acid synthase (FASN), and stearoyl-CoA desaturase 1 (SCD1). These pathways support membrane biogenesis and redox balance while also promoting metabolic flexibility, enabling adaptation to therapeutic and microenvironmental stresses. Emerging preclinical studies suggest that pharmacological inhibition of lipogenic enzymes, either alone or in combination with tyrosine kinase inhibitors, mTOR inhibitors, HIF-2α antagonists, or immune checkpoint blockade, may suppress ccRCC progression. However, most therapeutic data remain limited to preclinical models, and clinical validation is still lacking. This review synthesizes recent advances in molecular regulation and therapeutic targeting of DNL in ccRCC and discusses the challenges and future opportunities to improve mechanistic understanding and explore potential therapeutic applications.

## 1. Introduction

Globally, renal cell carcinoma (RCC) was the 14th most commonly diagnosed cancer and the 16th leading cause of cancer-related death, with 434,419 new cases and 155,702 deaths reported in 2022 [1]. RCC originates from the renal tubular epithelium and comprises three main histological subtypes: clear cell RCC (ccRCC), papillary RCC (pRCC) and chromophobe RCC (chRCC) [2]. Among them, ccRCC accounts for approximately 75% of all RCC cases [3,4]. Localized RCC is primarily managed through curative surgical resection; however, nearly 30% of patients eventually encounter disease recurrence following the initial treatment of localized tumors [5,6]. Systemic therapies are typically used for advanced metastatic disease or residual disease following surgery [5]. Over the past two decades, the therapeutic landscape for RCC has been revolutionized by immune checkpoint inhibitors (ICIs) targeting PD-1, PD-L1, and CTLA-4, particularly as this malignancy is notoriously resistant to conventional chemotherapy and radiotherapy [5,7]. While the 5-year relative survival for those with locoregional RCC has improved, reaching 72%, the prognosis for metastatic cases remains significantly less favorable, with a 15% survival rate [8].

Metabolic reprogramming, a hallmark of cancer, enables tumor cells to adapt to their microenvironment and sustain proliferation and therapeutic resistance through alterations in glucose, lipid, and amino acid metabolism [9]. The characteristic “clear” cytoplasmic phenotype of ccRCC reflects the accumulation of intracellular lipids and glycogen. This accumulation reflects profound metabolic reprogramming within tumor cells. Indeed, RCC, especially ccRCC, is widely recognized as a metabolic disease driven by genetic mutation-induced dysregulation of aerobic glycolysis, fatty acid (FA), and tryptophan and glutamine metabolism [10,11]. Lipids are essential for membrane structure, energy storage, and signal transduction, and their cellular levels are maintained through a dynamic balance between lipid synthesis, degradation, and utilization [9]. Therefore, regulation of fatty acid levels significantly influences tumor development via changes in de novo lipogenesis (DNL), FA uptake, and oxidation [12]. ccRCC exhibits dysregulated lipid homeostasis characterized by increased lipid synthesis and reduced lipid catabolism [13]. Consequently, cholesterol, FAs, and triglycerides accumulate within tumor cells. This metabolic reprogramming facilitates membrane biogenesis and tumor cell proliferation, while concurrently attenuating FA oxidation [14].

Alterations in DNL have been observed in various cancers, including breast [15], lung, and prostate cancers [16]. This metabolic dysregulation has prompted the investigation of DNL inhibitors, such as ATP-citrate lyase (ACLY), acetyl-CoA carboxylase (ACC), and FA synthase (FASN) inhibitors, as potential therapeutic agents in pre-clinical and clinical trials [17]. While current therapeutic strategies for ccRCC primarily target angiogenesis and immune checkpoints, accumulating evidence suggests that altered de novo lipogenesis (DNL) represents an additional metabolic vulnerability with potential therapeutic relevance. Recent reviews have summarized the broader roles of lipid metabolism in renal cancer progression and therapeutic resistance [18,19]. However, a focused synthesis specifically addressing ccRCC-selective DNL rewiring, upstream VHL/HIF-driven regulation, and translational therapeutic challenges remains limited.

In this review, we summarize current understanding of DNL regulation in ccRCC, with particular emphasis on VHL/HIF-driven lipogenic reprogramming, key DNL-associated enzymes, therapeutic targeting strategies, and current barriers limiting clinical translation. We further discuss how genetic alterations contribute to DNL dysregulation during ccRCC tumorigenesis and evaluate current evidence supporting therapeutic targeting of lipogenic pathways in ccRCC.

## 2. De Novo Lipogenesis Pathway and Physiological Regulation

Although lipogenesis and de novo lipogenesis (DNL) are closely related concepts, DNL specifically refers to the synthesis of FAs from non-lipid carbon sources, primarily glucose and glutamine [20]. Under physiological conditions, DNL is largely restricted to specific tissues, such as hepatocytes, adipocytes, and lactating mammary glands [21]. In contrast, many cancer cells aberrantly activate this pathway to sustain rapid proliferation, membrane biosynthesis, and metabolic adaptation, even in the presence of exogenous lipid sources [22].

### 2.1. De Novo Lipogenesis Pathway

The primary substrate for FA synthesis is cytoplasmic acetyl-CoA, which can be generated from either citrate or acetate [23]. Citrate is produced through the oxidation of glycolysis-derived pyruvate, in the TCA cycle, or via reductive carboxylation of glutamine-derived carbons. Bypassing the need for citrate, acetate can be directly incorporated into cytosolic acetyl-CoA through the activity of acetyl-CoA synthetase 2 (ACSS2) under metabolic stress, including hypoxia and lipid depletion [24]. Cytosolic citrate is converted to acetyl-CoA by ATP-citrate lyase (ACLY), providing substrate for acetyl-CoA carboxylase (ACC) [25]. ACC catalyzes the rate-limiting step of DNL, converting acetyl-CoA to malonyl-CoA. FASN then utilizes seven malonyl-CoA molecules and one acetyl-CoA to eventually generate palmitate (16:0), the principal saturated FA produced through DNL.

**Figure 1:**
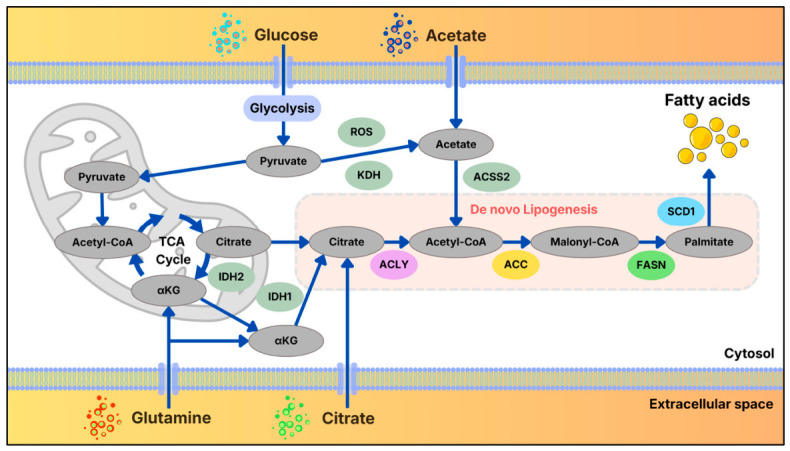
De novo lipogenesis pathway. De novo lipogenesis (DNL) integrates glucose, acetate, and glutamine metabolism. Glucose-derived pyruvate generates mitochondrial acetyl-CoA and citrate, which is exported to the cytosol and cleaved by ACLY to produce acetyl-CoA. ACC converts acetyl-CoA to malonyl-CoA, the first committed intermediate, and FASN catalyzes the synthesis of palmitate (C16:0). Palmitate is desaturated by SCD1 to yield monounsaturated FAs for membrane remodeling and lipid storage. Acetate contributes via ACSS2-derived acetyl-CoA, while under hypoxia or impaired citrate export, glutamine-derived α-ketoglutarate fuels lipogenesis through IDH1/2-mediated reductive carboxylation.

Palmitate can subsequently undergo desaturation through stearoyl-CoA desaturase (SCD) and fatty acid desaturase 2 (FADS2), generating the monounsaturated fatty acids (MUFAs) palmitoleate and sapienate, respectively [26] (Figure 1). In parallel, elongation of very-long-chain fatty acid (ELOVL) enzymes extends fatty acid carbon chains to generate structurally diverse lipid species. Although polyunsaturated fatty acids (PUFAs) can be synthesized from dietary essential fatty acids, the efficiency of conversion to bioactive PUFA species remains relatively limited [27]. These FAs are subsequently utilized for membrane biosynthesis, energy storage, and lipid signaling through their incorporation into complex lipid molecules such as phospholipids.

Importantly, several of these enzymes, including ACLY, ACC, FASN, and SCD1, are aberrantly upregulated in ccRCC and contribute to the characteristic lipid-rich phenotype of this malignancy.

### 2.2. Physiological Regulation of De Novo Lipogenesis

The regulation of DNL occurs through multiple coordinated layers that ensure tight control of lipid synthesis in response to cellular metabolic status. These regulatory mechanisms operate at several hierarchical levels, including direct modulation of enzymatic activity, post-translational modifications, and transcriptional control of lipogenic genes. Together, these mechanisms integrate metabolic signals derived from nutrient availability, hormonal stimulation, and cellular energy status. For clarity, the physiological regulation of DNL can therefore be broadly categorized into three major levels: allosteric regulation, covalent modifications, and transcriptional regulation, each of which contributes to the dynamic control of lipogenic flux.

#### 2.2.1. Allosteric Regulations

ACC activity is tightly regulated through allosteric mechanisms that coordinate lipogenic flux with cellular metabolic status. Citrate serves as a key activator by binding to a regulatory site distinct from the catalytic center, thereby promoting the transition to an active conformation [28,29]. ACLY integrates metabolic signals to coordinate acetyl-CoA production for lipid biosynthesis. Its activity is stimulated by citrate and glycolytic intermediates, including glucose-6-phosphate (G6P) and fructose-6-phosphate (F6P), thereby linking glucose metabolism to DNL [30,31]. Conversely, acetyl-CoA and oxaloacetate (OAA) provide feedback inhibition to restrain excessive lipogenic flux [30].

Long-chain fatty acyl-CoAs (LCFA-CoAs), including malonyl-CoA and palmitoyl-CoA, act as key negative regulators of DNL by direct inhibition of ACC and indirect activation of AMPK-dependent ACC phosphorylation [32]. Together, these feedback mechanisms prevent excessive lipid accumulation and maintain metabolic homeostasis.

#### 2.2.2. Covalent Modifications

Covalent modifications play a central role in regulating DNL by dynamically coordinating lipogenic enzyme activity with cellular energy status and metabolic demand. Among these regulatory pathways, AMP-activated protein kinase (AMPK) functions as a major suppressor of lipogenesis through coordinated phosphorylation of both metabolic enzymes and transcriptional regulators. AMPK directly phosphorylates ACC, thereby reducing its catalytic activity, while simultaneously modulating multiple lipogenic transcription factors, including Sterol Regulatory Element-Binding Protein 1c (SREBP1c), upstream stimulatory factor 1 (USF1), and carbohydrate-responsive element-binding protein (ChREBP) [33,34,35,36,37,38,39,40]. In addition, cAMP-dependent protein kinase (PKA) negatively regulates ACC activity through inhibitory phosphorylation [36]. Together, these signaling events function to re-strain excessive lipogenic flux during energy stress conditions.

ACLY activity is also regulated through phosphorylation-dependent mechanisms linking oncogenic and metabolic signaling to acetyl-CoA production. Kinases and phosphatases, including protein kinase B (AKT), PKA, glycogen synthase kinase 3 (GSK3), branched-chain α-keto dehydrogenase kinase (BDK), and protein phosphatase Mg^2+/^Mn^2+^ dependent 1 K (PPM1K), dynamically modulate ACLY activity and SREBP1 signaling, thereby coordinating lipid biosynthesis with cellular metabolic requirements [41,42,43,44,45]. In parallel, ACLY and other lipogenic enzymes are further regulated through proteolytic cleavage and ubiquitin-mediated degradation. Caspase-10-mediated ACLY cleavage and constitutively photomorphogenic 1 (COP1)-dependent fatty acid synthase (FASN) degradation represent additional mechanisms that suppress lipogenic activity under metabolic stress conditions [46,47].

Collectively, these covalent modifications establish a tightly coordinated regulatory network that dynamically controls DNL in response to nutrient availability, oncogenic signaling, and cellular energy balance.

#### 2.2.3. Transcriptional Changes

At the transcriptional level, DNL is regulated through an integrated network of nutrient-responsive transcription factors and nuclear receptors that coordinate lipogenic gene expression according to cellular metabolic demands [48]. Among these regulators, sterol regulatory element-binding proteins (SREBPs), particularly SREBP1c, function as the principal transcriptional drivers of lipogenesis. Under low-cholesterol or hyperinsulinemic conditions, SREBP precursors dissociate from insulin-induced gene (INSIG)-mediated retention in the endoplasmic reticulum (ER) and are transported by SREBP-cleavage-activating protein (SCAP) to the Golgi apparatus for proteolytic activation by membrane-bound transcription factor site 1 and 2 proteases (MBTPS1/2) [49,50,51]. The cleaved N-terminal SREBP fragments subsequently translocate to the nucleus, where they induce transcription of lipogenic genes including ACLY, ACC, FASN, and SCD1 [25].

Carbohydrate-responsive element-binding protein (ChREBP) represents another major transcriptional regulator linking glucose availability to lipogenesis. Elevated intracellular glucose metabolites such as glucose-6-phosphate (G6P) and xylulose-5-phosphate promote protein phosphatase 2A (PP2A)-mediated dephosphorylation of ChREBP, facilitating its nuclear translocation [52]. In the nucleus, ChREBP forms a heterodimeric complex with Max-like protein X (MLX) and activates lipogenic gene transcription through carbohydrate response elements (ChoREs). ChREBP signaling is further amplified through the induction of the more transcriptionally active ChREBPβ isoform [52].

This transcriptional network is further amplified by nuclear hormone receptors, notably liver X receptors (LXRs) and peroxisome proliferator-activated receptors (PPARs). Activated LXRs enhance lipogenic gene expression through direct transcriptional activation of SREBP1c and other DNL-associated enzymes [53,54,55]. Similarly, PPAR family members integrate FA-driven metabolic signals and regulate genes involved in lipid synthesis, transport, and metabolic flux through binding to peroxisome proliferator response elements (PPREs) [56,57,58,59,60]. Additional transcriptional regulators, including upstream stimulatory factor 1 (USF1), NF-I, NF-Y, and FOSB, further contribute to context-dependent regulation of SCD1 and other lipogenic enzymes [48,61,62,63,64,65,66].

**Figure 2:**
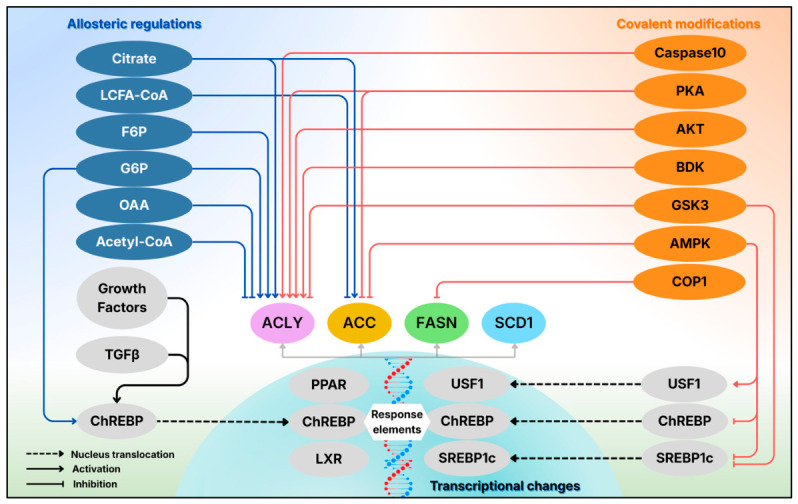
Multilayered regulations of de novo lipogenesis. DNL is controlled by coordinated allosteric, post-translational, and transcriptional mechanisms targeting key enzymes (ACLY, ACC, FASN, and SCD1). Metabolites such as citrate, F6P, and G6P stimulate, whereas OAA and LCFA-CoA inhibit lipogenic flux, with additional input from growth factor and TGFβ signaling. Enzyme activity is further regulated by phosphorylation via kinases, including AMPK, AKT, PKA, GSK3, and BDK, and by proteasomal degradation mediated by caspase-10 and COP1. At the transcriptional level, SREBP1c and ChREBP coordinate DNL gene expression under the control of upstream regulators such as LXR, PPAR, and USF1.

Collectively, transcriptional regulation of DNL involves a highly integrated network in which nutrient sensors, nuclear receptors, and hormonal signaling converge to coordinate lipogenic gene expression. Among these pathways, SREBP1c and ChREBP represent the principal transcriptional regulators governing DNL under nutrient-responsive and metabolically stressed conditions. All regulatory events associated with DNL are depicted in Figure 2.

## 3. Dysregulation of De Novo Lipogenesis in ccRCC

In contrast to normal renal epithelial cells, ccRCC exhibits profound metabolic reprogramming characterized by extensive lipid accumulation. This phenotype reflects not only increased lipid uptake but also enhanced DNL and altered FA utilization [67]. Multiple studies have further demonstrated aberrant activation of key lipogenic enzymes and upstream regulatory networks in ccRCC, contributing to tumor progression, metabolic adaptation, and therapeutic resistance [68]. To better understand these alterations, this section summarizes dysregulation of DNL in ccRCC from two complementary perspectives: the altered expression of core lipogenic enzymes and the upstream regulatory mechanisms controlling their activity.

### 3.1. Key Lipogenic Enzymes Driving Metabolic Reprogramming in ccRCC

ACLY plays a vital role in connecting carbohydrate breakdown and FA production by transforming citrate, a product of the TCA cycle, into acetyl-CoA. Given that ccRCC cells, due to HIF-driven Pyruvate dehydrogenase kinase 1 (PDK1) activation, prioritize glucose conversion to lactate, they generate citrate through the reductive carboxylation of α-ketoglutarate derived from glutamine [69]. This citrate is then utilized by ACLY to synthesize acetyl-CoA, providing the necessary materials for FA synthesis. A study of 33 paired ccRCC and adjacent normal kidney tissue samples revealed elevated ACLY mRNA and protein levels in the tumor, and this increase was associated with higher T stage and Fuhrman grade [70]. Investigations into ACLY’s role showed that knockdown with small interfering RNA led to a decrease in ccRCC proliferation and migration, and an increase in apoptosis [70]. Furthermore, ACLY suppression led to reduced ccRCC lipid droplet formation and EMT inhibition, potentially interacting with immune infiltration and the mTORC1 pathway [71].

ACC, which governs the rate of FA synthesis by producing malonyl-CoA from acetyl-CoA, demonstrates increased mRNA expression in ccRCC relative to normal kidney tissue. This upregulation of ACC has been identified as a predictor of poor overall survival in patients with ccRCC [72]. Notably, a large-scale multi-omic investigation of ccRCC tumors highlighted that elevated ACC protein levels are among the strongest protein markers associated with unfavorable survival rates [73]. Studies have demonstrated that targeting ACC, either through direct allosteric inhibition with 5-tetradecyloxy-2-furoic acid (TOFA) or by indirect activation of AMPK, can impede ccRCC cell growth. Specifically, TOFA was observed to limit cell growth in both ccRCC and pRCC by disrupting mTOR signaling and causing cell-cycle arrest at the G2/M phase [74].

Palmitate, the end-product of DNL, is generated by FASN through the condensation of seven malonyl-CoA and one acetyl-CoA. This enzyme, which demonstrates altered expression in ccRCC and various other cancers, plays multiple roles. In a cohort of 120 patients (93% with ccRCC) undergoing nephrectomy, immunohistochemical analysis revealed that elevated FASN levels were positively correlated with higher tumor stage, lymph node involvement, and the presence of distant metastases. Furthermore, increased FASN expression was found to predict poorer cancer-specific survival [75,76]. Another study indicated that FASN overexpression is associated with aggressive ccRCC phenotypes, including increased cell proliferation, migration, and lipid storage, and contributes to metabolic disruption. Moreover, elevated FASN mRNA correlates with visceral adiposity, a significant indicator of poor survival in ccRCC [77]. Given its role in ccRCC, FASN is considered a promising target for treatment, and numerous drugs designed to inhibit its activity are under development. Among these, C75, a well-characterized small molecule, has demonstrated efficacy in various ccRCC cell lines by suppressing cell proliferation and migration, inducing cell death, and cell cycle arrest [78].

SCD1 facilitates the conversion of saturated FAs (SFAs), like palmitate, into MUFAs, which are then utilized in the creation of glycerophospholipids and sphingolipids for cellular membranes, as well as in the production of signaling molecules. Elevated levels of SCD1 were observed in a majority of ccRCC patients, demonstrating statistically significant correlations with patient age, TNM staging, regional lymph node involvement (pN stage), Fuhrman grading, and tumor dimensions. Multivariate analysis identified SCD1 expression as an independent predictor of decreased overall survival [79]. Studies have identified increased SCD1 expression throughout the progression of ccRCC. Disrupting SCD1 through genetic knockdown or pharmacological inhibitor resulted in decreased proliferation and increased apoptosis in both in vitro and in vivo ccRCC models. Mechanistically, this effect appears to be mediated through the induction of endoplasmic reticulum stress, as evidenced by gene array, qPCR, and protein analysis of SCD1-inhibited or knockdown samples [80]. The key enzymes involved in DNL in ccRCC are summarized in Table 1.

### 3.2. Regulation of Key De Novo Lipogenesis Enzymes in ccRCC

DNL in ccRCC is controlled by a complex regulatory network that integrates transcriptional programs, metabolic enzyme activity, epigenetic modulation, and protein stability mechanisms. Accumulating evidence indicates that multiple oncogenic and metabolic regulators cooperate to reprogram lipid metabolism in ccRCC, thereby promoting lipid droplet deposition, tumor growth, and adaptation to metabolic stress. These regulators influence DNL at different levels, including transcriptional activation of lipogenic genes, modulation of metabolic flux, chromatin and RNA modifications, as well as post-translational control of key metabolic proteins. To provide a clearer mechanistic overview, the regulators involved in DNL are categorized into several functional groups based on their primary modes of action, including transcriptional regulators, metabolic enzymes, epigenetic regulators, and factors controlling ubiquitination and protein stability. Among currently identified regulatory networks, the VHL/HIF–SREBP axis represents the central and most functionally validated driver of lipogenic reprogramming in ccRCC. In contrast, several recently identified regulators appear to modulate DNL indirectly through AMPK-, SREBP-, inflammatory-, epigenetic-, or protein-stability-related mechanisms, but their functional relevance remains supported mainly by limited preclinical evidence. Therefore, in the following sections, we distinguish well-established regulatory pathways from emerging mechanisms that require further validation across independent ccRCC models.

#### 3.2.1. Transcriptional Regulators

Transcriptional control serves as a primary driver of metabolic reprogramming and aberrant lipogenic phenotype in ccRCC. Rather than functioning as isolated metabolic pathways, DNL in ccRCC is tightly integrated into upstream oncogenic networks. Emerging evidence suggests that the HIF-2α–METTL3–TCF7L2 axis may contribute to hypoxia-associated lipogenic reprogramming in ccRCC. Under hypoxia, HIF-2α transactivates the methyltransferase methyltransferase-like 3 (METTL3), enhancing m6A methylation and stability of transcription factor 7-like 2 (TCF7L2) mRNA. Stabilized TCF7L2 then complexes with β-catenin, triggering canonical Wnt signaling to drive lipogenic programs [81,82]. Moreover, cell-cycle machinery is directly incorporated to fuel tumor malignancy. The cell-cycle activator E2F1 acts as a potent lipogenic driver by enhancing SREBP1 transcription. This E2F1–SREBP1 axis is vital for ccRCC progression; its disruption suppresses FA synthesis, halts proliferation, blunts the epithelial–mesenchymal transition (EMT), and attenuates metastasis in vivo, consistent with patient data linking high E2F1/SREBP1 expression to poor survival [83,84].

This oncogenic regulatory network is further amplified by Krüppel-like factor 6 (KLF6) and mediator of RNA polymerase II transcription subunit 15 (MED15), both of which converge on SREBP activation to sustain lipid homeostasis. KLF6 has been reported to be upregulated in metastatic ccRCC and modulate lipid flux via dual mechanisms: direct transcriptional control of lipogenic genes, and indirect activation of SREBF1/2 via PDGFB–mTOR signaling [85,86]. Similarly, MED15 has been proposed to function as a transcriptional coactivator linking hypoxia signaling to SREBP-associated lipogenic programs. Induced directly by HIF-2α, MED15 physically interacts with SREBPs to enhance lipid biosynthetic enzyme expression, while simultaneously stimulating SREBP1/2 processing via the PLK1–AKT pathway [87,88].

Collectively, these findings support an SREBP-centered transcriptional network in which hypoxia signaling, cell-cycle progression, and growth factor pathways contribute to DNL regulation in ccRCC. However, compared with the VHL/HIF–SREBP axis, several recently identified transcriptional regulators remain supported primarily by mechanistic preclinical studies.

#### 3.2.2. Signaling Pathways Controlling De Novo Lipogenesis

DNL in ccRCC is tightly orchestrated by a complex network of signaling pathways that integrate extracellular stimuli, metabolic stress, and organelle-associated signaling with core lipogenic machinery. A recent study has identified GPX8 as a potential regulator linking ER stress adaptation to lipogenic signaling in ccRCC independently of VHL status [89]. Mechanistically, GPX8 modulates nicotinamide N-methyltransferase (NNMT) via the IL6–STAT3 axis; its disruption triggers AMPK activation, thereby suppressing DNL, triglyceride esterification, and ccRCC growth [90]. Similarly, ancient ubiquitous protein 1 (AUP1), the ER- and lipid droplet-associated protein [91,92], is highly upregulated in advanced ccRCC, where its expression correlates with advancing clinical stage. AUP1 accelerates DNL and fuels tumor progression, at least in part through suppression of the AMPK–ACC pathway [93]. This oncogenic signaling network is further amplified by chemokine-like receptor 1 (CMKLR1) [94] and sphingosine kinase 1 (SPHK1). While CMKLR1 has been implicated in sustaining SREBP-associated lipogenic signaling and tumor growth in ccRCC [95], SPHK1 upregulation has been associated with poor prognosis and enhanced lipogenic phenotypes in ccRCC. However, the precise mechanistic relationship between SPHK1 signaling and DNL regulation remains incompletely understood and requires further validation [96,97].

In contrast to these lipogenic drivers, dihydrolipoamide branched chain transacylase E2 (DBT) has been proposed to function as a metabolic suppressor of lipogenic signaling [98,99]. It is frequently silenced in ccRCC by METTL3-mediated m6A epitranscriptomic modification. In its intact form, DBT interacts with annexin A2 (ANXA2) at its lipoyl-binding domain to activate Hippo signaling, thereby restricting YAP nuclear translocation and suppressing downstream lipogenic gene expression [100].

Taken together, these findings suggest that multiple signaling pathways may contribute to DNL regulation in ccRCC. However, the strength of evidence varies substantially among regulators. AMPK–ACC and SREBP-associated signaling remain the most consistently validated regulatory nodes, whereas GPX8-, SPHK1-, CMKLR1-, and DBT-associated pathways should currently be interpreted as emerging mechanisms requiring broader functional and translational validation.

#### 3.2.3. Metabolic Enzymes Modulating De Novo Lipogenesis

Beyond canonical lipogenic enzymes, metabolic rewiring in ccRCC further enhances DNL by redirecting carbon flux and adapting substrate utilization under hypoxic and nutrient-stressed conditions. Several metabolic enzymes have also been implicated in lipid metabolic rewiring in ccRCC, including oxoglutarate dehydrogenase-like (OGDHL), a mitochondrial matrix enzyme [101]. ccRCC tissues exhibit a marked reduction in OGDHL; its depletion, driven by FTO-mediated m6A demethylation, stabilizes the transcription factor AP-2 alpha (TFAP2A) by preventing its ubiquitination. Stabilized TFAP2A then directly transactivates FASN and activates oncogenic ERK signaling to fuel lipid deposition [102]. Similarly, the loss of mitochondrial acetoacetyl-CoA thiolase (ACAT1), an enzyme vital for ketone dynamics and isoleucine degradation [103], has been associated with advanced clinical progression. Restoring ACAT1 activates AMPK signaling to suppress FA flux, thereby inhibiting ccRCC proliferation and metastasis [104].

This metabolic rewiring extends to the restriction of malonyl-CoA decarboxylase (MLYCD) [105]. Downregulation of MLYCD predicts unfavorable clinical prognoses in ccRCC, as its deficiency alters intracellular malonyl-CoA pools to expand lipid droplets, thereby reducing lipid-induced cellular stress and increasing resistance to ferroptosis [106]. For FA oxidation, ccRCC routinely silences the beta-oxidation enzyme hydroxyacyl-CoA dehydrogenase alpha subunit (HADHA) [107], where low expression significantly correlates with poor survival outcomes [108]. Restoration of HADHA expression suppresses lipogenic signaling and reduces tumor-associated lipid accumulation in experimental ccRCC models [109].

Beyond mitochondrial deactivation, cytoplasmic enzyme alterations actively drive lipogenesis, exemplified by the aldehyde dehydrogenase 9A1 (ALDH9A1) [110]. Diminished by FTO-induced m6A demethylation, the loss of ALDH9A1 protein disrupts the cytoplasmic sequestration of nucleophosmin 1 (NPM1), promoting its nuclear accumulation to repression of IQ motif containing the GTPase-activating protein 2 (IQGAP2). The resulting hyperactivation of the AKT–mTOR pathway triggers SREBP1 maturation [111].

Collectively, these findings suggest that metabolic enzyme dysregulation contributes to lipogenic reprogramming in ccRCC by altering carbon flux, fatty acid metabolism, and stress adaptation pathways. Many of these mechanisms ultimately converge on SREBP- and AMPK-associated signaling networks to support lipid accumulation and metabolic adaptation. However, the precise contribution of several recently identified metabolic regulators to ccRCC progression requires broader functional and clinical validation in ccRCC.

#### 3.2.4. Epigenetic Regulators

Epigenetic modifications append an additional layer of metabolic control by modulating the transcriptional landscape of lipogenic genes in cancer. A potential epigenetic regulator of lipogenic transcriptional programs in ccRCC is SETD8 (also known as PR-Set7 or SET8), a nucleosome-specific methyltransferase that coordinates genomic functions including DNA replication, mitosis, and gene expression via histone H4 lysine 20 monomethylation (H4K20me1) [112]. Clinical data reveal that SETD8 is markedly overexpressed in ccRCC tumors, with its abundance correlating with accelerated progression, robust metastasis, increased lipid storage, and adverse patient survival outcomes. At the molecular level, SETD8 is post-translationally stabilized by the deubiquitinase USP17. Once stabilized, SETD8 deposits H4K20me1 marks at specific genomic loci to directly enhance the transcriptional activity of SREBP1. Consequently, genetic downregulation of SETD8 disrupts this lipogenic programming, effectively inhibiting cell proliferation and metastatic potential in ccRCC models [113]. Altogether, epigenetic regulation of DNL in ccRCC relies on SETD8 stability, which forces chromatin remodeling to activate SREBP-centered programs and promote tumor progression.

#### 3.2.5. Ubiquitination and Protein Stability

Post-translational modifications, particularly ubiquitination-dependent protein degradation, play an important role in regulating the abundance and stability of lipogenic proteins in ccRCC. The E3 ubiquitin ligase tripartite motif containing-21 (TRIM21) exemplifies this protein-level checkpoint [114]. Deeply downregulated in ccRCC tumors, a loss of TRIM21 strongly correlates with unfavorable patient survival. Mechanistically, TRIM21 physically targets SREBP1 for ubiquitination and subsequent proteasomal degradation, thereby suppressing SREBP1-associated lipogenic signaling [115]. Mirroring this tumor-suppressive circuitry, the multi-functional E3 ubiquitin ligase ring finger protein 20 (RNF20) balances chromatin architecture via histone H2B monoubiquitylation [116,117]. In ccRCC, RNF20 functions as a critical metabolic barrier, restricting oncogenic lipid-reprogrammed phenotype by attenuating SREBP1c-dependent lipogenesis and pituitary tumor transforming gene 1 (PTTG1) [118].

To sustain a lipogenic tumor phenotype, ccRCC actively breaks this degradative machinery by silencing specific E3 ligases or overexpressing stabilizing kinases. The phospholipid-remodeling enzyme lysophosphatidylcholine acyltransferase 1 (LPCAT1) is highly overexpressed in ccRCC tissues, where its abundance tracks with aggressive pathological traits, advanced TNM stages, and shortened overall survival [119,120]. Driven directly by hypoxic activation via HIF-2α, elevated LPCAT1 suppresses the NF-κB pathway to downstream downregulate F-Box/WD Repeat-Containing Protein 7 (FBXW7), a crucial lipid-regulating E3 ubiquitin ligase. Because FBXW7 normally targets the core DNL enzyme ACLY for degradation, the HIF-2α–LPCAT1-mediated suppression of FBXW7 stabilizes ACLY protein levels, promoting FA production [121]. Beyond avoiding ligase-mediated destruction, ccRCC cells exploit the constitutively active serine/threonine kinase CK2 [122]. By maintaining the stability of SCD1, CK2 supports intracellular MUFA homeostasis in ccRCC cells [123].

Collectively, ubiquitin-dependent protein stability mechanisms contribute to the regulation of lipogenic signaling in ccRCC by modulating the abundance of key DNL-associated proteins. Loss of tumor-suppressive E3 ligases and stabilization of lipogenic enzymes collectively support pathological lipid accumulation and tumor progression.

#### 3.2.6. Mitochondrial Dynamics Regulator

Mitochondrial fusion and fission dynamics have emerged as critical spatial regulators that rewrite the lipogenic architecture of ccRCC. Mitofusin 2 (MFN2), an essential outer mitochondrial membrane protein, plays a central role in this organelle-level regulation, which is essential for maintaining mitochondrial homeostasis [124]. MFN2 expression is significantly downregulated in ccRCC tumors. Overexpressing MFN2 suppresses mitochondrial fragmentation and actively blunts ccRCC cell proliferation, migration, invasion, and xenograft tumor growth. Mechanistically, MFN2 suppresses HIF-2α signaling while promoting degradation of ACC and FASN proteins through autophagy-dependent and -independent mechanisms [125].

In summary, ccRCC rewires DNL through multiple interconnected regulatory layers: gene transcription, epigenetic modifications, signaling pathways, metabolic enzymes, protein stability, and mitochondrial dynamics. However, the strength of evidence differs considerably among these regulators. Currently, the most robust functional and translational evidence supports the VHL/HIF–SREBP axis and its downstream lipogenic enzymes, whereas many recently identified regulators remain supported primarily by mechanistic or exploratory preclinical studies and require broader validation. To provide a clear overview of this diverse landscape, Table 2 summarizes these regulatory layers, their molecular mechanisms, downstream targets, and associated clinical implications in ccRCC.

## 4. Mutation-Driven Alterations in De Novo Lipogenesis in ccRCC

**Figure 3:**
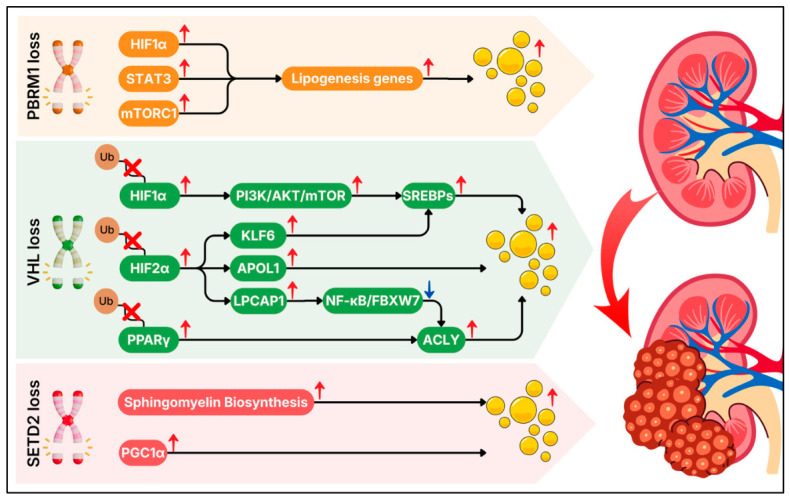
Mutation-driven events driving alterations in DNL in ccRCC. Loss of VHL results in constitutive stabilization of HIF-1α and HIF-2α, promoting PI3K–AKT–mTOR–SREBP signaling and activation of lipogenic enzymes including ACLY, ACC, FASN, and SCD1. VHL deficiency also stabilizes PPARγ and ACLY, thereby enhancing lipogenic flux. HIF-2α further reinforces lipid metabolic reprogramming through APOL1-, LPCAT1-, and KLF6-associated transcriptional networks. Additional mutations involving PBRM1 and SETD2 further potentiate lipogenic and metabolic rewiring programs in ccRCC (red upward arrows: activation, blue downward arrows: inhibition).

Comprehensive genomic analyses from the Catalog of Somatic Mutations in Cancer (COSMIC) and the Cancer Genome Atlas (TCGA) have identified recurrent alterations in multiple tumor suppressors and metabolic regulators in ccRCC, including VHL, PBRM1, SETD2, BAP1, KDM5C, PTEN, mTOR, PIK3CA, and TP53 [73,126,127]. Among these, VHL loss represents the dominant molecular event and serves as a major driver of hypoxia-associated metabolic reprogramming in ccRCC. These genetic alterations collectively provide important mechanistic insight into the dysregulation of de novo lipogenesis (DNL) and lipid metabolic adaptation during ccRCC progression (Figure 3).

Loss of von Hippel–Lindau (VHL) function constitutes the foundational event in ccRCC, driving extensive hypoxia-associated metabolic reprogramming. Under normoxic conditions, VHL mediates the degradation of HIFs (HIF-1α and HIF-2α), whereas VHL inactivation results in their constitutive stabilization and sustained transcriptional activity [128]. This persistent HIF signaling underpins tumor growth by promoting proliferation, adaptation to metabolic stress, and extensive metabolic rewiring. One major metabolic consequence of persistent HIF activation is the induction of anabolic lipid metabolism. Particularly, HIF-1α promotes activation of the PI3K/AKT/mTOR/SREBP signaling axis, thereby enhancing cholesterol ester synthesis and lipogenic gene expression [129]. In addition, persistent HIF signaling ultimately enhances expression of multiple downstream lipogenic enzymes, including ACLY, ACC, FASN, and SCD1, thereby sustaining anabolic lipid metabolism in ccRCC. In parallel, VHL directly regulates lipid metabolism through ubiquitination of PPARγ. Loss of VHL stabilizes PPARγ, enhances ACLY transcription, and promotes lipogenic metabolic reprogramming associated with mitochondrial dysfunction [130].

HIF-2α acts as a key downstream effector of VHL loss by coordinating transcriptional programs associated with lipid metabolic reprogramming in ccRCC. It directly regulates APOL1 expression through both promoter binding and the lncRNA LINC02609, thereby contributing to lipid-associated gene expression and tumor progression [131]. HIF-2α also induces LPCAT1, which suppresses NF-κB signaling and reduces expression of the E3 ubiquitin ligase FBXW7, leading to stabilization of ACLY [121]. In addition, HIF-2α sustains KLF6 expression through activation of a super-enhancer, integrating transcriptional control of metabolic genes with mTOR-driven activation of SREBF1 and SREBF2 [86]. These observations are particularly relevant given the recent clinical emergence of HIF-2α-targeted therapies in ccRCC, further supporting the translational importance of HIF-2α-driven metabolic reprogramming.

Although both HIF-1α and HIF-2α contribute to metabolic adaptation in ccRCC, accumulating evidence suggests partially distinct biological functions in lipid metabolic reprogramming. HIF-1α primarily promotes glycolytic adaptation and anabolic signaling under hypoxic conditions, whereas HIF-2α appears to function as the dominant oncogenic driver sustaining lipid storage, lipid remodeling, and maintenance of the lipogenic phenotype in ccRCC [132]. Several experimental and transcriptomic studies have further demonstrated stronger associations between HIF-2α activity and aggressive tumor behavior, metabolic plasticity, and therapeutic resistance in ccRCC [133]. Particularly, HIF-2α-dependent transcriptional programs involving APOL1, LPCAT1, and KLF6 support the persistent activation of lipogenic pathways downstream of VHL loss. Nevertheless, the precise mechanistic interplay between HIF-1α and HIF-2α in regulating DNL likely remains context-dependent and incompletely understood.

Beyond the VHL–HIF axis, recurrent mutations in chromatin regulators further potentiate metabolic dysregulation. Loss of PBRM1 amplifies HIF-1α- and STAT3-dependent transcription and promotes mTORC1 activation, reinforcing lipogenic gene expression programs [134,135]. Similarly, histone methyltransferase SET-domain-containing 2 (SETD2) deficiency drives metabolic reprogramming by enhancing de novo sphingomyelin biosynthesis [136] and promoting a shift toward oxidative phosphorylation and lipogenesis, potentially via PGC1α-dependent mechanisms [137]. Collectively, these genetic and epigenetic alterations converge to promote lipid metabolic reprogramming in ccRCC, with VHL loss–driven HIF activation functioning as the central regulatory axis.

## 5. Preclinical Therapeutic Targeting of De Novo Lipogenesis in ccRCC

Given the prominent lipogenic phenotype of ccRCC, pharmacological targeting of DNL has emerged as a potential metabolic therapeutic strategy. However, most currently available evidence remains preclinical, and ccRCC-specific in vivo validation is still limited. Among currently investigated approaches, SCD1 inhibition has demonstrated relatively stronger in vivo antitumor activity in ccRCC models, whereas evidence supporting ACLY and ACC inhibition remains largely restricted to in vitro studies.

### 5.1. Lipogenesis Inhibitors

Among currently available ACLY inhibitors, only a limited number have been directly evaluated in ccRCC models, whereas most preclinical evidence has been derived from other malignancies [17,138]. In ccRCC, hydroxycitric acid significantly suppresses cell proliferation, migration, and invasion in vitro, although no in vivo studies have yet been reported [71]. In contrast, extensive preclinical evidence from other cancer types demonstrates that ACLY inhibitors such as hydroxycitric acid, SB-204990, and BMS-303141 can suppress cancer stemness [139] and tumor growth in vitro and in vivo [140,141,142]. Additional agents, including bempedoic acid and morusin, have shown efficacy in hepatocellular carcinoma models through metabolic disruption and induction of oxidative stress [143,144]. However, early studies with hydroxycitric acid and SB-204990 were confounded by systemic effects such as weight loss and uncertain target specificity, and the liver-specific activation of bempedoic acid complicates the interpretation of its anticancer effects [145]. Therefore, the therapeutic relevance of ACLY inhibition in ccRCC still requires dedicated in vivo validation.

Functional evidence for targeting ACC in ccRCC remains limited but suggestive, as the allosteric inhibitor TOFA induces apoptosis and cell cycle arrest through suppression of PI3K/AKT/mTOR signaling [74]. Beyond ccRCC, multiple ACC inhibitors, including ND-646, TOFA, and ND-654, have demonstrated robust antitumor activity across cancer models by suppressing lipogenesis, inducing endoplasmic reticulum stress, and enhancing therapeutic responses [146,147,148,149]. PF-05175157 further exhibits antiproliferative effects in breast cancer models and synergizes with other targeted agents [150]. However, no clinical trials have yet evaluated ACC inhibitors in oncology, necessitating further assessment of their therapeutic efficacy and safety. Importantly, current evidence supporting ACC inhibition in ccRCC remains largely limited to in vitro studies, and dedicated in vivo validation is still lacking.

FASN represents one of the most extensively studied lipogenic targets in cancer metabolism; however, direct evidence supporting FASN inhibition in ccRCC remains comparatively limited. In ccRCC, the FASN inhibitor C75 suppresses cell proliferation, supporting a functional role for FASN in tumor growth [78]. Broadly, FASN inhibition via agents such as cerulenin, C75, and orlistat has demonstrated antitumor activity across multiple cancer types in both in vitro and in vivo models [151,152,153,154,155,156,157,158,159]. More selective inhibitors, including Fasnall, GSK2194069, IPI-9119, and TVB-3166, have further validated FASN dependency in diverse malignancies [153,155,156] with particular vulnerability observed in lipid-restricted environments such as brain metastases [160]. Early-phase clinical trials of TVB-2640 indicate favorable tolerability and emerging efficacy signals in solid tumors [153,157]. Nevertheless, earlier compounds were limited by toxicity, particularly weight loss, and clinical data remain sparse, underscoring the need for dedicated evaluation in ccRCC.

Compared with other lipogenic enzymes, SCD1 inhibition currently has relatively stronger functional support in ccRCC models, including evidence demonstrating impaired tumor growth, induction of ER stress, and enhanced sensitivity to targeted therapies. For example, the SCD1 inhibitor A939572 reduces proliferation and induces apoptosis, partly through endoplasmic reticulum stress, and exhibits synergistic effects with temsirolimus [80,123]. In vivo, T-3764518 reduces tumor burden in ccRCC xenograft models without significant toxicity [161]. Consistent with these findings, SCD1 inhibition across other malignancies, including colorectal, hepatocellular, and ovarian cancers, impairs proliferation, stemness, and tumor progression [162,163,164]. However, despite strong preclinical support, no SCD1 inhibitors have yet advanced to clinical trials in oncology, highlighting a key translational gap. Collectively, these findings position SCD1 inhibition as one of the most functionally validated DNL-targeting strategies currently available in ccRCC preclinical models.

### 5.2. Lipogenesis Inhibitors and Targeted Therapy

Combination strategies involving inhibitors of ACLY, ACC, FASN, and SCD1 have demonstrated synergistic antitumor activity in multiple preclinical cancer models through disruption of lipid metabolic adaptation. In ccRCC, the SCD1 inhibitor A939572 enhanced the antitumor activity of the mTOR inhibitor temsirolimus in A498 xenograft models by inducing lipid deprivation and unfolded protein response signaling [80].

Evidence from non-ccRCC malignancies further supports the therapeutic potential of combination metabolic targeting. For example, ACLY inhibitor BMS-303141 enhances Crizotinib efficacy in ALK-positive NSCLC via impaired FA supplement [165] and Sorafenib sensitivity in HCC in vivo [141], while ACC inhibitor ND-646 improves Carboplatin responses in NSCLC xenografts [146]. Additionally, the FASN inhibitor TVB-2640 is currently being evaluated in phase I clinical trials in combination with Taxane-based therapy or Enzalutamide in several advanced malignancies [166,167].

Collectively, these findings support the potential feasibility of combining DNL-targeting agents with TKIs or mTOR inhibitors. However, most supporting evidence currently remains preclinical, and clinical efficacy in ccRCC patients has not yet been established.

### 5.3. Lipogenesis Inhibitors and Immunotherapy

Emerging evidence suggests that lipogenesis inhibitors may influence tumor immunogenicity and responsiveness to immunotherapy, although currently available data remain limited and highly cancer-type specific. In MASH-associated hepatocellular carcinoma models, ACLY inhibition with EVT0185 reduced tumor burden as monotherapy and enhanced the efficacy of anti-PD-L1 and anti-VEGFR antibodies by promoting CXCL13 secretion, B-cell and plasma cell infiltration, and tertiary lymphoid structure formation [168]. These findings suggest that metabolic targeting may modulate antitumor immunity beyond direct tumor cytotoxicity.

Conversely, ACLY blockade with bempedoic acid or SB-204990 unexpectedly upregulates PD-L1 expression via PUFA peroxidation and cGAS-STING activation in hepatoma, pancreatic ductal adenocarcinoma, and melanoma models [169]. Although this response may enhance sensitivity to anti-PD-L1 therapy, it may also contribute to immunosuppressive signaling and T-cell dysfunction. To date, no studies have reported ACC, FASN, or SCD1 inhibitors combined with immunotherapy in ccRCC. Therefore, additional ccRCC-specific studies are required to clarify the immunologic consequences and therapeutic relevance of DNL inhibition in this disease.

Importantly, the translational development of DNL-targeting therapies in ccRCC remains at an early stage. While several lipogenic inhibitors have shown anti-tumor activity in diverse cancer models, only a limited number have been specifically investigated in ccRCC (Table 3). Furthermore, differences in tumor metabolic plasticity, lipid uptake dependency, and tumor microenvironmental adaptation may limit direct extrapolation from other malignancies. Therefore, additional ccRCC-focused preclinical and clinical studies are required to determine the therapeutic relevance of DNL inhibition in this disease.

## 6. Therapeutic Rationale for Targeting De Novo Lipogenesis in ccRCC

ccRCC is characterized by a lipid-enriched cytoplasm that reflects active metabolic reprogramming rather than a passive histological feature. Loss of VHL and subsequent HIF activation promote DNL through SREBP-dependent upregulation of key enzymes [170]. This metabolic shift supports membrane biosynthesis, redox balance, and adaptation to therapeutic stress, thereby contributing to tumor maintenance and drug resistance [171]. In this context, inhibition of lipogenic pathways is being explored as a strategy to disrupt metabolic homeostasis and impair tumor adaptation to hypoxia and oxidative stress [14].

Rather than acting in isolation, lipogenic enzymes are embedded within a broader VHL–HIF–SREBP regulatory network that integrates metabolic and epigenetic control. This perspective has led to interest in combinatorial strategies that co-target metabolic enzymes alongside upstream signaling pathways or transcriptional regulators to achieve more sustained metabolic suppression. In particular, combining lipogenesis inhibitors with tyrosine kinase inhibitors, mTOR inhibitors or HIF-2α antagonists may help limit adaptive resistance by concurrently targeting angiogenic signaling and metabolic dependencies [172]. In parallel, increasing evidence suggests that altered lipid metabolism contributes to an immunosuppressive tumor microenvironment, raising the possibility that modulation of DNL could influence anti-tumor immunity and improve responses to immune checkpoint therapies [173]. Taken together, current evidence suggests that DNL represents a metabolic dependency in at least a subset of ccRCC tumors. However, most supporting evidence remains preclinical, and the relative contribution of DNL compared with alternative lipid acquisition pathways in human ccRCC remains incompletely understood.

## 7. Future Perspectives and Translational Challenges

Despite encouraging preclinical findings, several major challenges currently limit the clinical translation of DNL-targeting strategies in ccRCC. One important concern is the potential toxicity associated with systemic inhibition of lipogenic enzymes, as DNL is also required for normal metabolic homeostasis in tissues such as the liver, adipose tissue, and immune cells. Indeed, early-generation inhibitors targeting ACLY and FASN were associated with adverse effects, including weight loss and metabolic disturbances [145,174,175]. Beyond toxicity-related concerns, adaptive metabolic rewiring represents another major barrier limiting the long-term efficacy of DNL-targeting therapies. Tumor metabolic plasticity may attenuate the efficacy of DNL inhibition, as cancer cells can compensate through compensatory activation of lipid uptake, fatty acid oxidation, or utilization of exogenous lipid sources [176].

In addition to therapeutic efficacy and metabolic adaptation, accurate patient stratification remains another critical challenge for clinical translation. ccRCC exhibits substantial metabolic heterogeneity, and not all tumors may depend equally on DNL. Biomarker-guided approaches incorporating expression of lipogenic enzymes such as FASN or SCD1, HIF-2α activation status, lipidomic profiling, or metabolic imaging may therefore be necessary to optimize patient stratification and therapeutic response.

These limitations collectively suggest that DNL inhibition alone may be insufficient for durable therapeutic responses in ccRCC. Given the complex metabolic adaptability of ccRCC, combination therapeutic strategies may prove more effective than DNL inhibition alone. In particular, combining DNL-targeting agents with currently established therapies, including VEGF-targeted tyrosine kinase inhibitors, HIF-2α inhibitors, mTOR inhibitors, or immune checkpoint inhibitors, may enhance antitumor efficacy while overcoming metabolic compensation mechanisms. Furthermore, preclinical evidence suggesting synergistic interactions between SCD1 inhibition and mTOR blockade supports the feasibility of metabolism-based combination approaches in ccRCC [80]. An additional emerging area of interest involves the interplay between DNL and ferroptosis sensitivity in ccRCC. Because lipid composition and fatty acid saturation influence ferroptotic vulnerability, future studies investigating how DNL-targeting strategies modulate ferroptosis may provide novel therapeutic opportunities and combination treatment approaches.

Collectively, although DNL-targeting strategies represent an emerging therapeutic opportunity in ccRCC, substantial translational challenges remain before clinical implementation can be achieved. Future studies integrating mechanistic biology, biomarker-guided patient selection, and rational combination therapies will be essential to determine the clinical applicability of targeting lipogenic metabolism in ccRCC.

## Figures and Tables

**Table 1:** Key enzymes involved in de novo lipogenesis in ccRCC.

Enzyme	Function	Evidence in ccRCC	References
ACLY	Converts citrate to acetyl-CoA for FA synthesis	Upregulated in ccRCC; correlates with higher stage and grade. Knockdown inhibits proliferation, migration, and lipid droplet formation	[69,70,71]
ACC	Converts acetyl-CoA to malonyl-CoA, the rate-limiting step of lipogenesis	Increased expression predicts poor survival. Pharmacologic inhibition suppresses tumor growth and induces G2/M arrest	[72,73,74]
FASN	Catalyzes the synthesis of palmitate from acetyl-CoA and malonyl-CoA	Overexpressed in ccRCC; associated with metastasis and poor survival. Inhibition reduces proliferation and induces apoptosis	[75,76,77,78]
SCD1	Converts saturated fatty acids into MUFAs	Frequently overexpressed; associated with advanced stage and poor prognosis. Inhibition reduces proliferation and induces apoptosis	[79,80]

**Table 2:** Molecular regulators controlling de novo lipogenesis in ccRCC.

Effect on DNL	Functional Category	Protein	Mechanism	Refs.
Activation	Epigenetic regulator	SETD8	Transcriptional activation of SREBP1 by monomethylating the 20th lysine of the H4 histone	[113]
	Signaling regulator	GPX8	AMPK deactivation through upregulating IL6/STAT3/NNMT pathway	[90]
		AUP1	Inhibiting AMPK phosphorylation	[93]
		SPHK1	Enhancing lipogenesis protein expression via an unknown mechanism	[97]
		CMKLR1	Regulating SREBP activation	[95]
	Transcriptional regulator	TCF7L2	Transcriptional activation of DNL genes through TCF7L2/β-catenin/Wnt pathway	[82]
		MED15	Coactivator of SREBPs and activation of SREBPs through the PLK1/AKT axis	[88]
		KLF6	Enhancing lipogenesis gene transcription and activation of SREBFs via the PDGFB-mTOR signaling pathway	[86]
		E2F1	Transcriptionally activating SREBP1	[84]
	Ubiquitination	LPCAT1	Inhibiting ACLY degradation via the NF-κB/FBXW7 pathway	[120,121]
		CK2	Maintaining SCD1 stability	[122,123]
Inhibition	Metabolic enzyme	ALDH9A1	Reducing SREBP1 maturation by NPM1-IQGAP2-AKT axis	[111]
		OGDHL	Deactivating FASN transcription by increasing TFAP2A ubiquitination	[102]
		ACAT1	Regulating DNL through the AMPK/ACC pathway	[104]
		MLYCD	Reducing malonyl-CoA content	[106]
		HADHA	Downregulation of ACLY and FASN	[108,109]
	Mitochondrial regulator	MFN2	Enhancing ACC and FASN degradation	[125]
	Signaling regulators	DBT	Inhibiting ACC and FASN transcription through the ANXA2/YAP axis-regulated Hippo pathway	[100]
	Ubiquitination	TRIM21	Regulating SREBP1 protein stability	[114,115]
		RNF20	Inhibiting the SREBP1c-PTTG1 axis	[118]

**Table 3:** Preclinical studies evaluating DNL-targeting therapies in ccRCC.

Target	Inhibitor	ccRCC Model	Combination Therapy	Key Findings	Refs.
ACLY	Hydroxycitric acid	Cell line	None	Suppressed proliferation, migration, and invasion of ccRCC cells	[71]
ACC	TOFA	Cell line	None	Induced apoptosis and cell cycle arrest via suppression of the PI3K/AKT/mTOR pathway	[74]
FASN	C75	Cell line	None	Inhibited tumor cell proliferation	[78]
SCD1	A939572	Cell line	None	Reduced proliferation and induced apoptosis through ER stress-related mechanisms	[80,123]
		Cell line and xenograft	Temsirolimus	Synergistically enhanced antitumor activity compared with monotherapy	[80]
	T-3764518	Xenograft	None	Reduced tumor burden without substantial toxicity	[161]

## Data Availability

No data was used for the research described in this article.
